# Impact of the COVID-19 pandemic: The perceptions of health professions educators

**DOI:** 10.15694/mep.2020.000142.2

**Published:** 2020-10-06

**Authors:** Richard Hays, Barbara Jennings, Trevor Gibbs, Julie Hunt, Kerrie McKay

**Affiliations:** 1 James Cook University; 2 Norwich Medical School; 3 Norwich Medical School; 4 Association for Medical Education in Europe; 5 College of Veterinary Medicine; 6 College of Veterinary Medicine

**Keywords:** Medical education, COVID-19, pandemic, innovation, learning technology

## Abstract

This article was migrated. The article was marked as recommended.

What are health professions educators doing during the COVID-19 pandemic? A search of articles in MedEdPublish on the topics of COVID-19 revealed 39 articles published in the first 3 months of the pandemic. Topics included curriculum adaptation, guidelines for using technology, assessment adaptation, impact on students, faculty and career development, and conference adaptation. There was significant overlap among articles, particularly those discussing teaching, learning, and assessment practices. Common themes were adaptation, innovation, remote delivery, flexibility in the face of a pandemic, and how to continue to educate and graduate competent health professionals. All articles were descriptive, and none included data describing efficacy, likely due to the short timeline since the pandemic’s inception. Additional study is necessary to produce evidence for the teaching and assessment adaptations described. Some changes are likely to persist longer-term and may outlast the pandemic itself.

## Background

The COVID-19 pandemic is a major event that has disrupted almost all aspects of life since January 2020. Health professions education is no exception, because it operates within the healthcare system, universities, and the broader community. Health care has had to switch rapidly to focus on both public health prevention strategies and the acute care of patients with serious COVID-19 infections. Education providers, like all sectors of society, have been subject to community-wide strategies introduced to limit the spread of disease. Additional personal hygiene requirements, social distancing, and restrictions on mobility and travel have resulted in community ‘lockdown’. With fewer patients, clinical teachers, students and professional staff at teaching campuses, all teaching, learning and assessment activities are affected. On-line content delivery has increased, clinical placements have been suspended or altered and assessment practices have had to change.

One additional change evident to editors of medical education journals is that academics seem to have had more time to write about their experiences. This journal has received substantially more manuscripts than normal. About 5 months into the pandemic, we have published 38 papers with either ‘COVID-19’ or ‘pandemic’ in the title or as the main subject. Contributions have come from a wide range of participants, including those in leadership roles, teachers, examiners and learners, and from many nations. MedEdPublish may be particularly suited to provide a window on what is current, because the journal has a relatively rapid publication cycle and publishes all manuscripts that meet ethical and formatting standards (about 90% of all submissions), allowing the community of practice to decide their value ( Hays, 2016). We prioritised the processing of these papers because of the high level of interest in ways of responding to the major disruption. The result is a compilation of early papers that describe how health professions educators managed through the initial stages of this evolving crisis, allowing a synthesis to be made of responses to the disruption. Here we provide a brief summary of strategies reported around the world.

## Method

A search for articles on the MedEdPublish website was conducted using ‘COVID-19’ and ‘pandemic’ as the primary search terms in the fields of ‘title’. The date of the final search was 30 ^th^ June, almost exactly 3 months after the first COVID-19 article was published on 23rd March. While the closing date is arbitrary, the period spans the initial outbreak of the pandemic and the rapid responses to the global disruption. A simple descriptive analysis was conducted to categorise articles by type and broad topic content, focusing on strategies for adapting to challenges, rather than simply describing them. All of the articles were set primarily in medical programs, with five including an interprofessional viewpoint across health professions’ education programs. Each paper was subjected to a ‘light touch’ thematic analysis to understand the common topics discussed in the health professions education community as the pandemic unfolded.

## Results

The 38 articles were spread across categories - ‘Personal view or opinion piece’ (15), ‘Practical tips and/or guidelines’ (13), ‘New educational method or tool’ (6), ‘Letter’ (2) and “Case study’ (2). None were research papers, probably reflecting the short time frame from action to publication. Many articles covered more than one topic, particularly the more general overview articles. For example, the use of technology was commonly mentioned in adapting most teaching, learning and assessment activities. The most common topics were the use of technology (28), curriculum or assessment adaptation (26), impact on undergraduate and postgraduate teaching (17), faculty and/or career development (7) and conference adaptation (3). The summary data for the articles and letters are presented in Table 1.

**Table 1.** A summary of 38 articles published in MedEdPublish during the first wave of the COVID-19 pandemic.

Type of MEP Manuscript	First Author & Location of Authors	Title of Article	Themes Discussed				
			TEL	Curriculum/Assessment Adaptation	Impact on UGT & PGT	Faculty Support & Career Development	Conferences & Scholarly Activity
**Personal View or Opinion Piece**	Alexander, UK	All hands on deck: early graduation of senior medical students in the COVID-19 pandemic		X	X		
	Alrefaie, Egypt & Saudi Arabia	Monitoring Online Learning During COVID-19 Pandemic; Suggested Online Learning Portfolio (COVID-19 OLP)	X	X			
	Arandjelovic, Australia	COVID-19: Considerations for Medical Education during a Pandemic		X	X		
	Cecilio-Fernandes, Brazil & UK	The COVID-19 pandemic and the challenge of using technology for medical education in low and middle income countries	X			X	
	Dyer, Barbados	Intimate Partner Violence: Using Standardized Patients to Improve Trauma-Informed Care in the era of the Covid-19 Pandemic			X		
	Fernandez-Altuna, Mexico	Experience of the biggest Med School in Mexico during the COVID-19 pandemic	X	X	X		
	Goh, Singapore & UK	A vision of the use of technology in medical education after the COVID-19 pandemic	X	X	X		
	Goh, Singapore & UK	Rethinking scholarship in medical education during the era of the COVID-19 pandemic		X			
	Hamad, Saudi Arabia	“To teach is to learn twice” Added value of peer learning among medical students during COVID-19 Pandemic	X				X
	Iwai, US	Transition to Virtual Reflection: Narrative Medicine during COVID-19	X	X	X		
	Johnson, US	Residents’ Perspectives on Graduate Medical Education during the COVID-19 Pandemic and Beyond	X	X			X
	Margolis, US & Uruguay	The Extended Congress: Reimagining scientific meetings after the COVID-19 pandemic	X				
	McKimm, UK & Australia	Health Professions’ Educators’ Adaptation to Rapidly Changing Circumstances: The Ottawa 2020 Conference Experience	X				X
	Sabzwari, Pakistan	Rethinking Assessment in Medical Education in the time of COVID-19		X	X		
	Woywodt, UK	COVID-19 - the ultimate disruptor?	X		X		
**Practical tips and/or guidelines**	Eachempati, Malaysia & India	Ten maxims for out of class learning to outclass the academic challenges of COVID-19	X		X		
	Fawns, UK	Challenging assumptions about “moving online” in response to COVID-19, and some practical advice	X	X	X	X	
	Foulds, Canada	From Spark to Flame -- Radical Innovations from Cataclysmic Events in Medical Education	X	X	X	X	
	Kachra, Canada	Practical tips for faculty development workforce training under pressure in the time of COVID-19 pandemic		X		X	
	Neufeld, Canada	Twelve tips to combat ill-being during the COVID-19 pandemic: A guide for health professionals & educators				X	
	Raja, UK	How to utilise your time effectively during the Covid-19 pandemic	X		X		
	Reyna, New Zealand	Twelve Tips for COVID-19 friendly learning design in medical education	X	X	X		
	Samarasekera, Singapore	Response and Lessons Learnt Managing the COVID-19 Crisis by School of Medicine, National University of Singapore	X	X	X		
	Sanders, UK, the Netherlands, Singapore, Germany, Oman, US, Canada, and Australia	Twelve tips for rapidly migrating to online learning during the COVID-19 pandemic	X	X			
	Taha, UAE & Saudi Arabia	Curriculum delivery in Medical Education during an emergency: A guide based on the responses to the COVID-19 pandemic	X	X			
	Taylor, UAE & UK	Transformation to learning from a distance	X	X		X	
	Wadi, Saudi Arabia & UAE	The assessment clock: A model to prioritize the principles of the utility of assessment formula in emergency situations, such as the COVID-19 pandemic		X			
	Wong, Singapore	Redesigning team-based learning facilitation for an online platform to deliver preclinical curriculum: A response to the COVID19 pandemic	X	X			
**New education method or tool**	Blythe, UK	Online Graduation of Doctors During the COVID-19 Pandemic	X		X		
	Khan, UK	An adaptation of Peyton’s 4-stage approach to deliver clinical skills teaching remotely	X	X			
	Mendes Chiloff, Brazil	Volunteering in medical school during the pandemic: a solution for teaching		X	X		
	Posner, Canada & US	Virtual reality videos for training and protocol dissemination during a pandemic	X		X		
	Sa-Couto, Portugal	How to use telesimulation to reduce COVID-19 training challenges: A recipe with free online tools and a bit of imagination	X	X			
	Sudhir, Dubai	Adapting to the need of the hour: Communication skills simulation session using an online platform during COVID-19	X	X			
**Case Study**	Boursicot, Singapore & Australia	Conducting a high-stakes OSCE in a COVID-19 environment		X		X	
	Veasuvalingam, Malaysia	Falling back on technology mindfully during COVID-19 pandemic: NUMed campus experience	X	X			
**Letter**	Niburski, Canada	A corona virus tracker for clinicians and students: Assessing education during an evolving phenomenon	X				
	Shimizu, Japan	More than adaptation: why we carried out faculty development on assessment in the middle of a pandemic	X	X			

MEP=MedEdPublish, TEL=Technology Enhanced Learning, UGT=undergraduate teaching and training, PGT=postgraduate teaching and training

There were examples from several schools in different countries that documented how faculty responded quickly and successfully to adapt to the rapidly evolving situation. Approaches were similar, and there has not yet been sufficient time for institutions to evaluate the impact of their interventions. The key to success appeared to be leadership and communication involving all stakeholders ( Samarasekera *et al.*, 2020). Lectures are now online, the use of simulation has increased and strategies for reducing the mixing of patients, students and staff for teaching and assessment are being developed. A common view is that health professions’ education will continue to use these strategies, at least to a substantial extent, even after ‘stability’ returns.

*Technology use.* Several articles addressed the need to scale up technology use, both using existing software and developing new software. Challenges for lower income countries were acknowledged ( Cecilio-Fernandes *et al.*, 2020), and the differential access to infrastructure between nations was explored. Faculty and students’ access to and proficiency with technology was also noted as being heterogenous. Authors provided practical tips about the use of blended learning with the right balance of synchronous and asynchronous online learning and teaching ( Eachempati *et al*., 2020; Sanders *et al.*, 2020).

*Curriculum adaptation.* Various degrees of blended learning, with some reliance on virtual learning environments, are now widespread and have probably allowed programs to continue, albeit quite differently. Almost all articles referred to students having to rely more on online learning methods, which was more socially isolating. This seems to be more straight-forward for more didactic methods, as lectures are relatively easy to broadcast and team-based learning can take place in online ‘rooms’ ( Wong *et al.*, 2020). Clinical teaching is much harder to adapt due to safety and narrower clinical workload. Placements were cancelled or deferred, except in some cases for more senior students, so innovation became essential. One interesting innovation is the use of simulated patients for communication skills teaching via live streaming ( Sudhir *et al.*, 2020). Virtual reality methods, where technology was available, were thought to be a potential growth area as part of a broader vision to extend the use of technology in medical education ( Goh and Sandars, 2020:2).

*Assessment adaptation.* Arguably, assessment, particularly clinical assessment, has suffered the greatest impact. While there were only four papers on adapting assessment, common themes emerged. The strongest message is that the forced change in assessment practices is an opportunity to improve assessment practice into the future. Assessment may become more programmatic, more reliant on many more methods and assessment events, and less reliant on large, high-stakes events ( Wadi *et al.*, 2020; Sabzwari, 2020). The recording and monitoring of assessment may become more important, with more sophisticated technology to support e-portfolios (Alrafiae *et al.*, 2020). While OSCEs can be modified to improve safety ( Boursicot *et al.*, 2020), will they continue at all, given the potentially high risks of transmission at crowded examination centres where patients, students and examiners from many places are thrown together?

*Faculty/career development.* Two more attributes seem to have been added to the list of desirable attributes for faculty members. One is a much deeper expertise in education methods, particularly those required to support remote teaching, learning and assessment ( Kachra and Ma, 2020). The unrealistic expectation that teachers can be rapidly pushed online without support and training was explored by Fawns *et al.*, 2020. The other is flexibility and a capacity to change education strategies rapidly ( Shimizu *et al.*, 2020). How to achieve this on a large scale is not addressed, although specific training in technology and managing disruption may become more frequent. The impact of surrounding community supports, such as childcare arrangements, public transport and the potential to maintain working from home are likely to remain prominent issues.

*Impact on learners.* Students at one school created an App that tracked rapidly changing information on COVID-19 to both increase knowledge about and protect them from the disease ( Niburski and Niburski, 2020). Similar software was developed in many nations for both the professions and the general public. Postgraduate specialty trainees reported that the narrowing of clinical workload may have had a greater effect on their training because some services closed and clinical staff were re-directed ( Johnson and Blitzer, 2020). The outcome may be a delay in completion in several speciality programs. There was also a report of volunteering of undergraduate students (and faculty) to assist in managing the heavy workloads ( Mendes *et al.*, 2020), and one of early graduation of final year students to support the workforce ( Alexander *et al.*, 2020). The ‘rite of passage’ of graduation ceremonies was maintained through live streaming ( Blyth *et al.*, 2020).

*Conference adaptation.* One article reported the rapid adaptation that had to be made with little warning to the Ottawa 2020 conference that was held at the beginning of March, just as the pandemic was taking hold outside of China ( McKimm *et al.*, 2020). Only about 30% of registrants could attend, and social distancing was encouraged. Plenaries and workshops switched to online presentations with international live streaming of questions and discussion. This appeared to work well, particularly for plenaries and some workshops that were discussion based rather than ‘hands-on’ practical in nature. The heroes were the IT team and participants who connected at very unfriendly hours. Another paper reported the conversion to online methods for an international conference held at the end of March, when the pandemic had progressed further. There were several hundred registrants in 20 countries and the most likely outcome was cancellation. Instead, organisers spread sessions over 4-8 weeks to allow for engagement through local and technology-mediated discussions while registrants continued to work ( Margolis *et al.*, 2020). These blended conference delivery approaches may become part of all future conferences.

## Discussion

What has emerged from this experience of the most severe global disruption in about 80 years (the second World War may have been a similar scale) may best be described as shared experiences and similar responses, with some genuine innovations and a need for further educational development, evaluation and research. What lessons can be learned? Some changes to health care and health professions education may remain for some time, perhaps becoming permanent. Further global disruptions, although rare in terms of human lifespans, are likely at some stage. Other, less severe disruptions may be more common. Telehealth and telehealth professional education may become more common and important.

As interesting as this collection of papers is, we are left with a series of questions that need to be answered for the community of practice to thrive. Should all lectures be recorded without audiences and be available as podcasts and videos? Will some students demand seated classes as part of their educational experience? Will medical educators embrace the tools in their virtual learning environments to enhance the design and effectiveness of their online lessons? How sustainable are large, high stakes written and clinical examinations? Should we move to more frequent, smaller assessment activities of greater breadth? Although simulation and virtual reality will develop further, how can immersion in real health care be maintained? How can we remain in a state of preparedness and ability to respond rapidly to future disruptions? How will faculty development evolve? Will working patterns remain more flexible and, if so, how will this affect career development? Will large international conferences survive? How will professional networks develop and thrive if ‘corridor’ conversations and debates over tea or coffee cannot take place? An issue that pervades most of these questions is: what is the value of social interaction in medical education?

These questions form a substantial agenda for research and debate as health professions’ education evolves. A quote from the business literature is apposite:

*’Vision without action is merely a dream. Action without vision just passes the time. Vision with action can change the world’* ( Barker, 1993).

## Limitations

This is a limited review of papers submitted to only one health professions education journal. All are self-report articles with no data or evaluation to guide judgments of effectiveness. The article therefore represents the thinking and responses formed on the run during a major global disruption, not a definitive review of how best to respond.

## Conclusion

Health professions educators had to respond quickly to the re-focusing of health care and healthcare staff to a narrower clinical caseload with tight infection control measures in highly stressed health care systems. The surrounding, community-wide measures such as enhanced personal hygiene measures, social distancing and travel restrictions, made campus attendance almost impossible. Programs switched rapidly to online methods where the technology was available. Clinical learning was cancelled or deferred, and where possible replaced by simulation, with the assistance of virtual reality. Learning and assessment were disrupted, most severely for those nearing graduation and in postgraduate specialty training. Amidst this turmoil, some interesting innovations have emerged. There is need for evaluation and research to produce evidence that guides the adoption of innovation in future teaching, learning and assessment practices. The recent disruption is an opportunity to improve health profession education beyond the duration of the pandemic. This article has focused on questions that need answers, so the focus should now be on evaluation of adaptation strategies.

## Take Home Messages

• Health professions’ education had to respond quickly to the rapidly unfolding COVID-19 pandemic
• Normal business was not possible
• Globally, responses appear to be similar
• The disruption has resulted in innovations that need evaluation
• Some of the changes are likely to persist as better ways are found

## Notes On Contributors

Professor Richard Hays, MBBS MD PhD is Professor of Remote Medicine and Health at James Cook University, Australia, who combines clinical practice with academic interests in curriculum and assessment design, implementation and evaluation. ORCiD: https://orcid.org/0000-0002-3875-3134

Dr Barbara Jennings, BSc (hons), PhD, PGCert (higher education practice), SFHEA is a Senior Lecturer at Norwich Medical School, UEA and MBBS-Genetics-Theme-Lead and Academic Lead for Faculty CPD and Module Lead for MClinEd (Contemporary Pedagogy module) in the Medical Education Department of Norwich Medical School, University of East Anglia, UK. Twitter account @GeneticsMBBS. ORCiD: https://orcid.org/0000-0003-3792-9182

Professor Trevor Gibbs is an Independent Consultant and Professor of Medical Education and Primary Care, and Professor of Medical Education, First Affiliated Hospital, Sun Yat-sen University, Guangzhou, China. He is President of the Association for Medical Education in Europe (AMEE). ORCiD: https://orcid.org/0000-0002-1776-6518

Dr Julie Hunt, DVM, MS, AFAMEE is Medical Director of the DeBusk Veterinary Teaching Center, College of Veterinary Medicine, Lincoln Memorial University, Harrogate, TN, USA. ORCiD: https://orcid.org/0000-0003-1927-432X

Kerrie McKay, BA (Hons) joined the Association for Medical Education in Europe in 2015 as part of the AMEE Secretariat Team based in Dundee, Scotland. Her primary role, as the MedEdPublish Administrator, is to support all aspects of the journal, including the Editorial Team, Editorial Board, Reviewing community and Authors. Kerrie was specifically involved in the processing of COVID-19 related manuscripts for this article from submission through to publication.

## Declarations

The authors are Editors or employees of MedEdPublish.
