# Supplementary material for: Impact of the COVID-19 pandemic: The perceptions of health professions educators
Source: MedEdPublish (2016). 2020 Oct 6;9:142. Originally published 2020 Jul 7. [Version 2] doi: 10.15694/mep.2020.000142.2 (PMC10702636; doi:10.15694/mep.2020.000142.2)
Supplement: Peer review history

# Reviewer report: Reviewer response for version 2

**DOI:** 10.21956/mep.20279.r31483

**Published:** 2020-12-05

**Authors:** Megan Anakin

**Affiliations:** 1 University of Otago

This review has been migrated. The reviewer awarded 4 stars out of 5 Thank you for inviting me to review the revised version of this article. Thank you for reorganising the first two sections of the article. The warrant for the article is now well-established. I am looking forward to a follow up article that examines further COVID-19 articles submitted to our journal.One new issue to bring to your attention is a minor error. In the abstract, you state 39 articles were sampled, however, in the methods and results only 38 articles are presented. In your reply, please let the reader know if this number is correct or not. Perhaps this error arose during the revision of this article. In the first version of the article, there is an article by Lee, How we taught medical students in a tertiary hospital during a pandemic present in the results but missing from the reference list and it is entirely omitted in the second version of this article.

Reviewer Expertise:

NA

No decision status is available

# Reviewer report: Reviewer response for version 2

**DOI:** 10.21956/mep.20279.r31482

**Published:** 2020-10-15

**Authors:** Süleyman Yildiz

This review has been migrated. The reviewer awarded 5 stars out of 5 I liked very much this article. Thanks for great summary of 39 articles about covid impact.The article is very well written with a good English. It provides excellent summary points with detailed tables and collection of references of medical education experiences during the COVID-19 pandemic in medical education. Conclusion part arises new questions and directs the readers to new researches. For example, the situation of medical education specifically poses an additional challenge during the pandemic, as this is a curriculum that is greatly based on clinical practice in many faculties. The question arises in my mind to what degree this situation affected medical students in clinical training as future physicians, as essential components of practical knowledge will not be available to learn via distance learning. Thanks for great job!

Reviewer Expertise:

NA

No decision status is available

# Reviewer report: Reviewer response for version 2

**DOI:** 10.21956/mep.20279.r31484

**Published:** 2020-10-07

**Authors:** Judy McKimm

**Affiliations:** 1 Swansea University

This review has been migrated. The reviewer awarded 4 stars out of 5 This artcile is a really helpful review of a series of rapidy published articles during the three months when the pandemic was taking hold across the world and imapcting on all aspects of HPEs life. The authors provide a good summary of both the artciles cited as well as the key findings from the literature. I recommend this artcile, not just as a summary of what has happened, but in providing a road map for what HPEs will have to cope with in the foreseeable future. It is salutory that already some of the predictions in the paper have come to fruition such as virtaul teaching and conferences, but it also reflects the inherent flexibility and adaptability of our community.

Reviewer Expertise:

NA

No decision status is available

# Reviewer report: Reviewer response for version 1

**DOI:** 10.21956/mep.19140.r27668

**Published:** 2020-10-03

**Authors:** Shaoting Feng

**Affiliations:** 1 The First Affiliated Hospital of Sun Yat-sen University

This review has been migrated. The reviewer awarded 4 stars out of 5 It is quite an interesting study on the perceptions of health professions educators.

Reviewer Expertise:

NA

No decision status is available

# Reviewer report: Reviewer response for version 1

**DOI:** 10.21956/mep.19140.r27660

**Published:** 2020-07-23

**Authors:** Annwyne Houldsworth

**Affiliations:** 1 HECL

This review has been migrated. The reviewer awarded 4 stars out of 5 Thank you very much for this timely article. This paper is extremely well written with a very high standard of English language. The paper provides a very valuable table with a collection of references of medical education experiences during the COVID-19 pandemic in medical education.The main themes of common findings that arise in the reference papers are well defined and described, giving an insight into some of the issues that have arisen during the pandemic crisis.There are a number of questions arise in the conclusion that could inform other medical educationalist researchers to initiate new investigations, researching into the future of medical education, including the evaluation of student experiences.Readers who are interested in viewing different medical education experiences and alternative methods of delivery will be able to search the table of references in this publication as a useful repository to seek new ideas.A small selection of examples of individual experience to highlight some of the generalized themes would be interesting to read.The addition of some educational theory background into curriculum design, assessment and the use of technology in medical education would have given a better contextual introduction to the paper.A table of the examples of technology and software used by the reference papers would be an extremely helpful addition to the paper. An example that highlights the most interesting practical tips that the author appreciated would be most appreciated.A description of a small sample of suggestions and examples of assessment methods that aim to improve or innovate the student assessment, found in the papers referenced, would have been an edifying and helpful addition.

Reviewer Expertise:

NA

No decision status is available

# Reviewer report: Reviewer response for version 1

**DOI:** 10.21956/mep.19140.r27659

**Published:** 2020-07-16

**Authors:** Jessica Servey

**Affiliations:** 1 Uniformed Services University of the Health Sciences F. Edward Hebert School of Medicine

This review has been migrated. The reviewer awarded 4 stars out of 5 I think this was a succinct overview of several articles. I have found faculty and clinicians more willing to accept innovations manuscripts with limited outcomes during this pandemic to share experiences. I wonder if that will continue. My concern is the lack of long-term questions in the discussion that address the outcomes of health professions education -------- is there a point that disruption affects the career of a clinician? Is there a truly foundational knowledge, and more importantly, skill level that must be done in person. Are we assuming virtual is just as good with no long term data? (which interestingly many scholars vehemently advocate against prior to this) Have we gone from adaptability to acceptability with no actual outcomes? Do the multiple-choice tests equal clinical practice 5 years later? These are my questions and I am concerned decisions we are making now with less information may adversely affect careers later. As far as faculty, I am uncertain about workplaces and conferences. There are some intangible difficult to measure items about those to include connection and career enhancement theorized to decrease burnout. Overall I agree that there are so many questions this pandemic has caused in health professions education.

Reviewer Expertise:

NA

No decision status is available

# Reviewer report: Reviewer response for version 1

**DOI:** 10.21956/mep.19140.r27667

**Published:** 2020-07-10

**Authors:** Ben Canny

**Affiliations:** 1 Monash University

This review has been migrated. The reviewer awarded 4 stars out of 5 Thank you for this article and summary of the reports published in MeEdPublish about responses to the COVID-19 pandemic. While somewhat out of the scope of the article, COVID-19 does present the opportunity for the assumptions that underpin many of our educational practices to be unpicked and challenged. This should represent a valuable field of educational scholarship into the near future, as suggested by the authors.

Reviewer Expertise:

NA

No decision status is available

# Reviewer report: Reviewer response for version 1

**DOI:** 10.21956/mep.19140.r27664

**Published:** 2020-07-08

**Authors:** BALAJI ARUMUGAM

**Affiliations:** 1 TAGORE MEDICAL COLLEGE AND HOSPITAL

This review has been migrated. The reviewer awarded 4 stars out of 5 It’s a great pleasure to read the commentary on the perceptions of health professions educators with the review of 39 articles published under Mededpublish. Authors are appreciated for publishing such a wonderful work. The technology use with adaptation towards curriculum and assessment to impact the learners is a great task for the past three to four months. Lock down periods were initially taken as a relaxation time for many of us but as gradually the days went on, we were forced physically/mentally to get adapted for the situation. The colleges/medical schools were closed for almost more than 2 months without regular personal classes and were transformed into virtual classes. The syllabus can be covered but how to ensure that the students have become competent. Online / Virtual classes can be taught but the assessment has become a bigger task especially after the implementation CBME from 2019 onwards under medical council of India. Good work by the authors. It’s worth reading this commentary. Thanks

Reviewer Expertise:

NA

No decision status is available

# Reviewer report: Reviewer response for version 1

**DOI:** 10.21956/mep.19140.r27663

**Published:** 2020-07-08

**Authors:** Viktor Riklefs

**Affiliations:** 1 Karaganda Medical University

This review has been migrated. The reviewer awarded 5 stars out of 5 Very nice and timely publication. I was just going to look at MedEdPublish for COVID-19 articles as we plan for the next academic year, and email came in suggesting me to read this one. It saved me time from reading all 39 published articles by providing very good summary of main conclusions and directions for further development and innovation at the time of crisis.

Reviewer Expertise:

NA

No decision status is available

# Reviewer report: Reviewer response for version 1

**DOI:** 10.21956/mep.19140.r27665

**Published:** 2020-07-08

**Authors:** Megan Anakin

**Affiliations:** 1 University of Otago

This review has been migrated. The reviewer awarded 4 stars out of 5 Thank you for summarising recent articles related to the COVID-19 pandemic in MedEdPublish. To help the reader, the authors may wish to put the methods section after the background section so the warrant for the review is clearly established before the methods are described. The sample of 39 articles produced six themes reported in the results. I noted that they reflect the challenges and opportunities that my colleagues and I have experienced at our medical school in New Zealand. The questions raised in the discussion will be useful to stimulate conversations with colleagues and help us to think about how we might design research study that evaluates the strategies we have adopted during the disruption to our educational programmes.

Reviewer Expertise:

NA

No decision status is available

# Reviewer report: Reviewer response for version 1

**DOI:** 10.21956/mep.19140.r27662

**Published:** 2020-07-08

**Authors:** Poh-Sun Goh

**Affiliations:** 1 National University of Singapore

This review has been migrated. The reviewer awarded 4 stars out of 5 Thank you to the Editorial team for inviting me to review this paper. This paper is a timely, informative, and thought provoking snapshot of three months of published work in MedEdPublish, reflecting and describing the clinical and academic response to the COVID-19 pandemic by the authors of the published papers. The spectrum of published papers, their range, and depth (of discussion); as well as the audience of these papers (views - readership numbers, open reviews - number and range of comments, and citations - for example on Google Scholar) give readers of MedEdPublish, and medical education scholars impressions and insights on what is topical, of value, potentially useful and of impact within the themes and topics available, visible and accessible on this open access, rapid, post-publication peer reviewed online journal. Examples of analytics (above), both quantitative and qualitative, and reflection on both published ideas, and visible commentary/reviews and engagement by the readership of MedEdPublish I believe adds to, and extends the ideals of Educational Scholarship and supports our aspirations as Digital Scholars.I commend the leadership of AMEE and MedEdPublish for their vision and commitment to launch and sustain the MedEdPublish scholarship platform, as well as the extremely hardworking committed editorial and administrative team at MedEdPublish. The fact that the AMEE community has embraced, and uses the MedEdPublish platform, to both publish, as well as read and engage with the material is evidence of the value-add and impact that this platform has for the members of AMEE, and communities of practice and interest within the AMEE membership, as well as wider medical education community.

Reviewer Expertise:

NA

No decision status is available

# Reviewer report: Reviewer response for version 1

**DOI:** 10.21956/mep.19140.r27661

**Published:** 2020-07-07

**Authors:** Jenny Alexanders

**Affiliations:** 1 Teesside University

This review has been migrated. The reviewer awarded 4 stars out of 5 This is an insightful, thought evoking article in relation to healthcare education during the COVID crises. It provides a detailed account in the way in which healthcare education (especially within the placement setting) as had to make rapid adjustments in order to provide students with as much clinical experience when practicing within tighter caseloads and more stringent infection control practices. It highlights the challenges that assessments bring to educators and how being adaptable through a digital platform has provided some strategies. The questions towards the end of this article provide researchers with a springboard to which further research can be conducted. A very good read.

Reviewer Expertise:

NA

No decision status is available

# Reviewer report: Reviewer response for version 1

**DOI:** 10.21956/mep.19140.r27666

**Published:** 2020-07-07

**Authors:** Samar Ahmed

**Affiliations:** 1 Ain Shams University Faculty of Medicine

This review has been migrated. The reviewer awarded 4 stars out of 5 I like this article so much. I truly think the authors Dis a great job analyzing the transition. I would like to draw the attention of the authors to a An articles that was not mentioned and could be a great addition to this work. https://www.mededpublish.org/manuscripts/3079I would also propose that the conclusion and highlights Showcase more what the areas of innovation were that will remain sustainable after the end if this COVID time. Evaluation of the initiatives use especially planned long term impact assessment is a meeting ch needed domain in the future as indicated in the conclusion. Congratulations on the exceptional work.

Reviewer Expertise:

NA

No decision status is available
